# Establishment of the Normal Reference Range of Thrombelastogram among the Healthy Population and Pregnants in China

**Published:** 2019-05

**Authors:** Rong GUI, Xueyuan HUANG, Ming ZHOU, Shujuan OUYANG, Yunfeng FU, Hao TANG, Fengxia LIU, Rong HUANG, Meng GAO, Hang DONG, Yongjun WANG

**Affiliations:** 1. The Third Xiangya Hospital, Central South University, Changsha, Hunan, China; 2. Department of Hematology, Hunan Provincial People’s Hospital, The First Affiliated Hospital of Hunan Normal University, Changsha, China; 3. The Hunan Cancer Hospital, Changsha, Hunan, China; 4. The Second Xiangya Hospital, Central South University, Changsha, Hunan, China

**Keywords:** Thrombelastogram, Normal reference range, Healthy volunteers, Pregnant women, China

## Abstract

**Background:**

We aimed to establish the reference range of thrombelastogram (TEG) for Chinese healthy volunteers and pregnant women and analyze the influence factors.

**Methods:**

Blood samples were collected from healthy volunteers and pregnant women at five tertiary hospitals (the Third Xiangya Hospital of Central South University, the Second Xiangya Hospital of Central South University, Hunan Provincial People’s Hospital, Hunan Cancer Hospital and Changsha Central Hospital) in 2016. The effects of age, gender, blood type, and full-term pregnancy on the reference range of normal TEG for healthy volunteers and pregnant women were studied. The specificity of TEG in detecting coagulation disorder.

**Results:**

For healthy volunteers, the normal ranges of TEG parameters were as follows: R, 4.3–9.3 min; K, 1.2–3.2 min; α, 50.2–71.2°; MA, 54.1–71.3 mm; LY30: 0%–2.2%; CI, −3.8–2.4. At least one parameter exceeded the normal range specified by the manufacturer in 20.3% of the healthy volunteers; about 7.6% healthy volunteers were diagnosed as coagulation disorder by the above standards; the specificity of detection was 79.7%. There were significant differences in R, K, α, MA and CI between males and females (*P*<0.01). For pregnant women, the normal ranges of TEG were as follows: R, 3.9–7.5 min; K, 1.0–2.4 min; α, 57.6–74.9°; MA, 55.7–75.7 mm; LY30, 0%–0.56%; CI, −0.97–3.6. Pregnant women having O blood group had a dramatically prolonged R. Full-term pregnancy had no significant impact on TEG results.

**Conclusion:**

Compared with pregnant women having non-O blood group, those having O blood group had a dramatically prolonged R and showed greater tendency to hemorrhage during and after parturition.

## Introduction

Many diseases can lead to coagulopathy, such as trauma. Early diagnosis and treatment of coagulopathy are important for a better prognosis of the patients. Thrombelastography (TEG) is mainly used for perioperative monitoring of the coagulation status and is often used to guide perioperative decisions on blood transfusion, thrombolytic therapy and anti-coagulation therapy (1, 2). TEG-guided decision on blood transfusion can reduce unreasonable use of blood products in liver transplantation, thus lowering the total cost (3). Because of these features, TEG has broad application prospect in clinics.

Establishment of the normal reference range is an important factor influencing TEG result and its diagnostic value. The normal reference ranges are provided by foreign manufacturers of the reagents. Many studies have shown that the normal reference range varies with ethnic group and geographical differences (4, 5). About 8.5% of the healthy subjects were diagnosed as coagulopathy if the normal reference range provided by the manufacturer was used (6). This pointed to the importance of establishing the normal reference range of TEG for the specific population.

The normal coagulation balance is usually disrupted during pregnancy and many pregnant women are in hypercoagulable state (7, 8), which can increase the risks of thrombosis and thrombotic diseases. Therefore, monitoring the coagulation function during pregnancy is of high importance. Studies have recommended the use of TEG for monitoring hypercoagulation in pregnant women (9, 10). Every delivery room should have TEG to provide a quick understanding of the coagulation state of pregnant women and to formulate the treatment strategy (11).

However, the reference range of TEG for ordinary people is not fit for pregnant women. Pregnant women with an O blood group are faced with a higher risk of hemorrhage (12). Blood group may be associated with hemorrhage after parturition, but it remains uncertain whether the ABO blood group causes coagulation function changes in pregnant women. At different stages of pregnancy, the coagulation functions differed (13). But the effect of full-term pregnancy (gestational age>37 wk) on coagulation functions in pregnant women is little known. Investigating combined effect of blood group and full-term pregnancy on TEG is a better pathway to understand the coagulation functions in pregnant women.

The purpose of this study was to establish the reference range of TEG for local healthy volunteers and pregnant women and to analyze the influence factors.

## Materials and Methods

### Subjects and Screening criteria

Healthy volunteers and pregnant women from China who received physical examination at five tertiary hospitals (the Third Xiangya Hospital of Central South University, the Second Xiangya Hospital of Central South University, Hunan Provincial People’s Hospital, Hunan Cancer Hospital and Changsha Central Hospital) from January to December 2016 were included. All of them were of the Han ethnic group who lived in China for over 3 years. In addition, female volunteers who met the requirements were selected from healthy volunteers as the control group of pregnant women TEG reference range.

The informed consent was signed before test, and the research protocol was approved by Ethnics Committee of the hospital.

The inclusion criteria for healthy volunteers: 1) normal results of physical examination, auxiliary examinations and serologic tests; 2) not diagnosed as other diseases within 3 months; 3) not having taken any other drugs within 7 days before test. Exclusion criteria were as follows: 1) pregnant women, women during a menstrual period; 2) patients with hemorrhagic diseases or thrombotic diseases, cases who took anticoagulants and hormonal contraceptives; 3) patients who were diagnosed as other diseases within 3 months; 4) the volunteers who did not consent to participate in the study. Volunteers were included only if their results of the routine coagulation tests were normal.

The exclusion criteria for pregnant women were: 1) below 15 yr or above 45 yr of age; 2) a medical history of coagulopathy and/or thromboembolic disease; 3) anti-coagulation treatment and/or treatment with anti-platelet drugs during pregnancy; 4) the women who did not consent to participate in the study. The exclusion criteria for control were:1) below 15 yr or above 45 yr of age; 2) a medical history of coagulopathy and/or thromboembolic disease; 3) anti-coagulation treatment and/or treatment with anti-platelet drugs in the last 30 days; 4) have used hormonal contraceptives in the last 6 months; 5) are currently pregnant; 6) have undergone delivery or abortion in the last 6 months; 7) the women who did not consent to participate in the study.

### Methods

Venous blood was collected from the healthy volunteers and pregnant women for routine coagulation test and TEG. Pregnant women and control group also collected blood for blood type testing. Citrated kaolin-TEG was performed using the TEG 5000® Thrombelastograph analyzer (Hemostasis System, USA). All tests were finished within 2 h after sample collection. Values of PT, APTT, FIB, TT, INR, R, K, α-angle, MA, LY30 and CI were recorded.

Values of TEG parameters were compared against the normal reference ranges provided by the manufacturer. To further analyze the influence of the normal reference ranges provided by the manufacturer on clinical diagnosis, the subjects were divided into two types based on Kaufmann’s classification (14): 1) Hypercoagulable state: shortened R and K, and increased α and (or) MA (with at least 2 conditions satisfied); 2) hypocoagulable state: prolonged R and K, and decreased α and (or) MA (with at least 2 conditions satisfied). If over two parameters were abnormal but the results were contradictory, hypercoagulable or hypocoagulable state was diagnosed based on the main parameter or the significantly abnormal parameter. Those with only one abnormal parameter were diagnosed as no coagulopathy. In addition, the specificity of TEG was assessed, and the influence of age and gender on the normal reference range of TEG was discussed. At the same time, the influence of blood type, full-term pregnancy on pregnant women’s TEG reference range and the correlation of parameter between pregnant women’s TEG and routine coagulation function were analyzed.

### Statistical analysis

SPSS 19.0 software (Chicago, IL, USA) was used for statistical analysis. The normal reference ranges for each TEG parameter were determined using normal distribution method (x̄±1.96 s) and percentile method. Measurements obeying a normal distribution were expressed as mean±standard deviation. The means of two samples were compared using t-test. Multi-group comparison was performed using ANOVA. Measurements not obeying a normal distribution were expressed as M(Q_0_–Q_4_) and analyzed by rank sum test. *P*<0.05 was considered significant.

## Results

### Baseline information

A total of 998 blood samples were collected from the healthy volunteers. All volunteers were of the Han ethnic group and aged 42.05±18.29 yr old. In addition, 566 pregnant women were enrolled, with an age of 30.54±4.1 yr, and 68.1% of pregnant women were ≥37 wk pregnant. Overall, 264 female volunteers were enrolled in the control group with an age of 31.8±7.7 yr. In the pregnancy group and control group, there was no significant difference in the distribution of blood type.

### Establishment of normal reference ranges

For volunteers, the normal reference range for R was 4.3–9.3 min; the normal reference range for K was 1.2–3.2 min; the normal reference range for α-angle was 50.2–71.2°; the normal reference range for MA was 54.1–71.3 mm; the normal reference range for LY30 was 0–2.2%; the normal reference range for CI was −3.8–2.4. TEG test results showed that, compared with the control group, there were significant differences in TEG of pregnant women (*P*<0.001), showing hypercoagulable state. For pregnant women, the normal reference range for R was 3.9–7.5 min; the normal reference range for K was 1.0–2.4 min; the normal reference range for α-angle was 57.6–74.9°; the normal reference range for MA was 55.7–75.7 mm; the normal reference range for LY30 was 0–0.56%; the normal reference range for CI was −0.97–3.6 (Table 1).

**Table 1:** Normal reference ranges for TEG parameters in healthy volunteers, pregnant women and control group

***Variable***	***R(min)***	***K(min)***	***Α(°)***	***MA(mm)***	***LY30(%)***	***CI***
Healthy volunteers(n=998)						
x̄±SD or M(Q_0_–Q_4_)	6.8±1.3	2.2±0.5	61.2±5.6	62.7±4.4	0.2(0–0.6)	−0.7±1.6
Reference range	4.3∼9.3	1.2∼3.2	50.2∼71.2	54.1∼71.3	0.0∼2.2	−3.8∼2.4
Pregnant women(n=566)						
x̄±SD or M(Q_0_–Q_4_)	5.7±0.98	1.61±0.45	67.8±5.29	68.7±4.72	0.09±0.34	1.46±1.38
Reference range	3.9∼7.5	1.0∼2.4	57.6∼74.9	55.7∼75.7	0∼0.56	−0.97∼3.6
Control group(n=264)						
x̄±SD or M(Q_0_–Q_4_)	6.31±1.12	1.97±0.38	63.48±4.23	64.07±3.78	0.68±0.94	−0.56±1.21
Reference range	4.4∼8.3	1.4∼2.7	53.5∼70.9	57.8∼71.0	0∼3.52	−2.3∼2.0

We also compared TEG parameters between males and females. The results showed that except for LY30 (*P*=0.181), all other TEG parameters were significantly different between males and females (Table 2). Therefore, we established the normal reference ranges for TEG parameters for males and females separately (Table 3).

**Table 2:** Comparison of TEG parameters between males and females [x̄±SD or M(Q_0_–Q_4_)]

***Group***	***Case***	***R(min)***	***K(min)***	***α(°)***	***MA(mm)***	***LY30(%)***	***CI***
Males	622	7.0±1.2	2.3±0.5	60.0±5.7	61.8±4.3	0.1(0.0–0.7)	−1.1±1.5
Females	376	6.4±1.3	1.9±0.4	63.9±4.4	64.3±4.1	0.2(0.0–0.8)	0.0±1.4
*P* value		0.000	0.000	0.000	0.000	0.181	0.000

**Table 3:** Normal reference ranges for TEG parameters in males and females

***Group***	***Case***	***R(min)***	***K(min)***	***α(°)***	***MA(mm)***	***LY30(%)***	***CI***
Males	622	4.6∼9.4	1.3∼3.3	48.8∼71.2	53.4∼70.2	0.0∼2.2	−4.0∼1.8
Females	376	3.9∼8.9	1.1∼2.7	55.3∼72.5	56.3∼71.9	0.0∼2.3	−2.7∼2.7

Comparison showed that the K values of volunteers aged 20–29 yr old were much higher than those of other age groups; α-angle and MA were significantly lower than those of other age groups, while CI value was higher in volunteers aged 20–29 yr old. In addition, the LY30 values of volunteers aged above 50 yr old were much lower compared with other age groups. The R values of volunteers aged 30–39 yr old and 50–59 yr old were significantly higher compared with other age groups (*P*<0.05) (Table 4). These results proved that age did influence the coagulation function.

**Table 4:** Comparison of TEG parameters between different age groups [x̄±SD or M(Q_0_–Q_4_)]

***Age (yr)***	***Case***	***R(min)***	***K(min)***	***α(°)***	***MA(mm)***	***LY30 (%)***	***CI***
≤19	138	6.6±1.4	2.1±0.5**	62.4±5.0^#^	62.8±4.3***	0.3(0.1–1.2)	−0.5±1.8
20–29	168	6.4±1.3	2.4±0.5	58.2±6.1	61.1±4.0	0.4(0.1–1.0)	−1.0±1.5^§^
30–39	160	7.0±1.3*	2.2±0.4	60.3±4.4^#▾^	62.2±3.6	0.1(0–0.6)	−1.0±1.4^§^
40–49	182	6.9±1.1	2.2±0.5**	61.4±5.2^#^	62.9±4.2***	0.2(0–0.9)	−0.7±1.5
50–59	179	7.1±1.2*	2.1±0.5**	61.7±5.3^#^	63.1±4.3***	0(0–0.5)^▲^	−0.8±1.6
≥60	171	6.6±1.3	2.0±0.6**	63.2±6.1^#^	64.0±4.4***	0(0–0.5)^▲^	−0.2±1.7

Note:

*, **, *** and^#^indicate *p*<0.05 compared with the age group of 20–29; ^▾^and^§^ indicate *P*<0.05 compared with the age group ≥60; ^▲^ indicates *P*<0.05 compared with the age group ≤19

### Comparison against the normal reference ranges provided by the manufacturer

The established normal reference ranges were compared against those provided by the manufacturer. Except for LY30, the values of all other parameters exceeded the normal reference ranges provided by the manufacturer. About 20.3% (203/998) of the volunteers had at least one parameter exceeding the normal reference range; 56 and 20 volunteers had three and two abnormal parameters, respectively. According to Kaufmann’s classification, about 7.6% of the volunteers were diagnosed as coagulopathy (hypercoagulable state in 52 cases and hypocoagulable state in 24 cases).

Furthermore, 127 volunteers had one abnormal TEG parameter (abnormal R in 42 cases, abnormal K in 20 cases, abnormal α-angle in 34 cases and abnormal MA in 31 cases), These cases were considered as no coagulopathy.

None of the volunteers had contradictory TEG parameters, and none of them had coagulopathy (as indicated by routine coagulation tests). Thus, the specificity of the normal reference ranges provided by the manufacturer in diagnosing coagulopathy in the local healthy population was 79.7%.

### The effect of blood group and full-term pregnancy on TEG during pregnancy

Compared with non-O blood group, those having O blood group presented with a much higher R (*p*=0.01), while other TEG parameters were insignificantly different except for CI (Table 5). To discuss the effect of full-term pregnancy on the coagulation functions of pregnant women, coagulation functions were assessed by TEG at different stages of pregnancy (whether the gestational age was ≥37 wk). It was found that full-term pregnancy had barely any impact on TEG. LY30 seemed to vary at different stages of pregnancy, but only insignificantly (Table 5).

**Table 5:** Comparison of TEG parameters between different ABO blood type and duration of pregnancy [x̄±SD or M(Q_0_–Q_4_)]

***Variable***	***R(min)***	***K(min)***	***α(°)***	***MA(mm)***	***LY30(%)***	***CI***
Blood type						
O group(n=215)	5.91±0.86	1.61±0.37	67.62±4.91	68.66±3.8	0.08±0.22	1.31±1.14
Non-O group(n=351)	5.58±1.01	1.63±0.48	67.54±5.45	68.66±4.53	0.1±0.41	1.53±1.45
*P* value duration of pregnancy	0.01	0.83	0.87	0.99	0.76	0.28
<37 wk(n=170)	5.67±1.19	1.60±0.39	67.63±5.44	68.70±4.36	0.16±0.52	1.43±1.23
≥37 wk(n=396)	5.66±0.92	1.61±0.45	67.88±5.29	68.67±4.87	0.06±0.25	1.45±1.35
*P* value	0.91	0.88	0.80	0.65	0.22	0.92

### Correlation between TEG and routine coagulation test

Table 6 shows the correlation between TEG and routine coagulation test in 566 pregnant women. As seen from the table, PT correlated positively with α and negatively with K; APTT correlated positively with R and K, and negatively with α, MA and CI; FIB correlated positively with R and MA; PLT and INR correlated positively with α, MA and CI and negatively with K.

**Table 6:** Correlation between TEG and routine coagulation test

***Variable***	***R***		***K***		***α***		***MA***		***LY30***		***CI***	
	***r***	***P***	***r***	***P***	***r***	***P***	***r***	***P***	***r***	***P***	***r***	***P***
PT	−0.075	0.335	−0.179	0.021	0.171	0.028	0.088	0.257	−0.054	0.492	0.148	0.057
APTT	0.311	0.001	0.205	0.008	−0.193	0.013	−0.2	0.01	−0.038	0.623	−0.308	0.001
FIB	0.173	0.026	−0.11	0.157	0.038	0.631	0.223	0.004	−0.059	0.449	0.037	0.635
INR	−0.184	0.17	−0.185	0.017	0.199	0.01	0.185	0.017	−0.045	0.562	0.242	0.002
PLT	−0.106	0.175	−0.322	0.001	0.259	0.001	0.43	0.001	0.09	0.247	0.341	0.001

## Discussion

Normal reference range is an important factor influencing the TEG results. However, the normal reference ranges for TEG are specified by the foreign manufacturers of reagents, and they vary greatly from one region to another according to literature reports. Many normal reference ranges of TEG have been reported by different laboratories in foreign countries, based on a variety of populations, including normal subjects (6), pregnant women (9) and children (15). Our results showed that the specificity of the normal reference range provided by the manufacturer was 79.7% in the local healthy population, agreed with the results by Scarpelini et al (6). In addition, similar to other studies (16, 17), we found that the females had stronger coagulation function and a greater proportion of females were in hypercoagulable state as compared with males. We also detected the influence of age on TEG. As age increased, K tended to decrease, while α, MA and CI increased. This indicated higher probability of hypercoagulable state in elderly volunteers, but the difference was of no statistical significance. The sample size should be further enlarged to verify the conclusion. The normal coagulation balance is disrupted in pregnancy, and pregnant women are usually in a hypercoagulable state in spite of the compensatory mechanisms. Such hypercoagulable state will not persist, and a normal coagulation balance will be restored within 3–6 wk after parturition (18, 19). However, it is still necessary to monitor the coagulation functions in pregnancy. Along with the implementation of China’s two-child policy, China will witness a surge number of pregnant women. In this context, how to ensure the safety of pregnant women with limited medical care resources will be the primary concern.

Compared with routine coagulation test, TEG can reflect the overall coagulation functions, TEG has been widely applied to surgery, blood transfusion and ICU (20–22). TEG is also used in obstetrics in foreign countries, but it is rarely used in this field in China.

We detected TEG parameters in pregnant women and healthy volunteers. The results showed that pregnant women had a much lower R, K and LY30 than the healthy volunteers, while α, MA and CI increased significantly in the former. That is to say, the pregnant women were in a hypercoagulable state as revealed by TEG. Our TEG results in pregnant women completely agreed with the changes to the coagulation functions in pregnancy (8). The above findings indicated the feasibility of using TEG parameters to monitor the coagulation state during pregnancy. We further analyzed the effect of blood group and full-term pregnancy on TEG parameters. Compared with non-O blood group, those with O blood group had a dramatically prolonged R, indicating that the level of coagulation factors in pregnant women with O blood group was much lower than that in pregnant women with non-O blood group. This may explain why pregnant women with O blood group are more likely to suffer from hemorrhage after parturition. Monitoring the coagulation functions during and after parturition is more necessary for women with O blood group. All TEG parameters were not significant differences between full-term pregnancy and non-full-term pregnancy. However, a greater sample size is needed to verify our findings. The correlation between routine coagulation test and TEG was further analyzed. Except for LY30, all other TEG parameters correlated to routine coagulation test. These results were consistent with the MA and PLT count and function reported in another study (23). Our results demonstrated that TEG may serve as an alternative to routine coagulation test in pregnant women.

Our study is China’s first large-sample research concerning the establishment of the normal reference range of TEG about healthy population and pregnant women. However, our study had certain limitations: 1) The sample size was relatively small; 2) All included subjects were of Han ethnic group and some provinces. So the effect of ethnic group and geographic location on TEG was not fully considered. Our future work will be devoted to multi-center clinical trial that has a larger sample size and recruits healthy population and pregnant women of different ethnic group. On this basis, we will try to establish the reference range of TEG for Chinese healthy population and pregnant women.

## Conclusion

To better understand the coagulation functions and to guide blood transfusion and treatment, it is necessary to establish the reference range of TEG for the local healthy subjects and pregnant women. Moreover, compared with pregnant women having non-O blood group, those having O blood group had a dramatically prolonged R and showed greater tendency to hemorrhage during and after parturition. A close monitoring of coagulation functions is needed for pregnant women having O blood group.

## Ethical considerations

Ethical issues (Including plagiarism, informed consent, misconduct, data fabrication and/or falsification, double publication and/or submission, redundancy, etc.) have been completely observed by the authors.
